# An intelligent model to decode students’ behavioral states in physical education using back propagation neural network and Hidden Markov Model

**DOI:** 10.1186/s40359-024-01743-4

**Published:** 2024-05-06

**Authors:** Liyan Li

**Affiliations:** Sports Department, Luoyang Normal University, Luoyang, 471934 Henan China

**Keywords:** Physical education, HMM, Neural network, Behavior decoding

## Abstract

This paper highlights the need for intelligent analysis of students’ behavioral states in physical education tasks. The hand-ring inertial data is used to identify students’ motion sequence states. First, statistical feature extraction is performed based on the acceleration and angular velocity data collected from the bracelet. After completing the filtering and noise reduction of the data, we perform feature extraction by Back Propagation Neural Network (BPNN) and use the sliding window method for analysis. Finally, the classification capability of the model sequence is enhanced by the Hidden Markov Model (HMM). The experimental results indicate that the classification accuracy of student action sequences in physical education exceeds 96% after optimization by the HMM method. This provides intelligent means and new ideas for future student state recognition in physical education and teaching reform.

## Introduction

Given the increasing concern for health, physical education plays a pivotal role in school education. As a distinct form of education, it not only develops students’ physical fitness and motor skills, but also their teamwork, leadership skills, and overall literacy. However, with the development of physical education, traditional methods are no longer sufficient to meet the educational needs. Advanced techniques are required to enhance the effectiveness of physical education [1].

Behavioral-motor analysis, as an important research method for physical education, has been widely used in recent years. By observing and recording students’ behavioral states in physical education classes, behavioral-motor analysis can provide insight into students’ motor behavior characteristics, individual differences and motor skill levels, and provide teachers with a scientific basis for teaching [2]. In physical education tasks, student behavioral states include a variety of behavioral manifestations of students in the physical education classroom, such as movement execution, movement skill level, emotional state, and social interaction. The study of students’ behavioral states aims to understand the patterns of behavioral changes, the relationship between behavioral states and academic performance, and possible influencing factors in different teaching environments, so as to provide a scientific basis for optimizing physical education teaching methods and improving students’ learning outcomes [3]. In addition, behavioral-motor analysis can help teachers identify students’ learning difficulties and problems so that they can target instructional adjustments and optimization.

However, there are limitations in traditional behavioral movement analysis methods, such as less efficient processing of large amounts of data and more subjective analysis results. Therefore, more intelligent methods, such as decoding analysis and machine learning methods, are needed to perform more accurate and objective behavioral state processing analysis. Decoding analysis, through pattern recognition and data mining of student behavior data, can automate the analysis of student behavior states and generate scientific data reports to provide teachers with more accurate teaching references [4]. At the same time, machine learning methods can be used to learn and model a large amount of student behavior data, thus enabling intelligent prediction of student behavior states and personalized teaching [5].

Therefore, this study will be based on the decoding analysis method, through in-depth research and analysis of students’ behavioral states in physical education, aiming to explore a more intelligent approach to physical education, so as to improve the effectiveness and quality of physical education and promote the realization of students’ overall quality education. Through this study, it will provide new ideas and methods for physical education research and practice, and make positive contributions to physical education reform and development, the specific contributions are as follows:

- Recognition and analysis of typical movement data from physical education courses using hand rings and extraction of statistical features of inertia data
- The BPNN model was trained by obtaining the data and the results obtained were further optimized using the HMM model
- According to the proposed BPNN-HMM model, four typical action sequences in physical education were classified, and their overall recognition rate reached more than 96%, which provides new ideas for future behavioral state analysis of physical education.

The rest of the investigation is organized as follows. In “Related works” section, related works for physical education using machine learning and decoding methods are introduced; in “A BPNN-HMM-based state analysis and processing model for sports behavior” section, the established model using BPNN-HMM is illustrated; “Experimental results and analysis” section describes the experiment and result analysis; In “Discussion” section, we discuss the results and the development direction for In “A BPNN-HMM-based state analysis and processing model for sports behavior” section, the established model using BPNN-HMM is illustrated; “Experimental results and analysis” section gives the experiment and result analysis; In “Discussion” section, we discuss the result and the development direction for the intelligent physical education. The conclusion is given at last.

## Related works

### Behavioral state research based on machine learning and decoding analysis

Decoding analysis methods are a class of methods used for model modeling and state identification of sequential data, and are commonly used to process time-series data. Commonly used decoding analysis methods include: hidden Markov models, Markov chains, dynamic time regularization, maximum entropy Markov models, and conditional random field methods, which have a wide range of applications in the prediction of behavioral sequences and related time series analysis [6].

Behavioral state research based on machine learning and decoding analysis has gained much attention in recent years. By collecting data from multiple sources, such as sensor data, image data, and voice data, researchers use machine learning and decoding analysis techniques to analyze and make inferences about the behavioral state of students, users, or other entities. Take the study by Smith et al. [7] as an example, they modeled and analyzed students’ behavioral states during learning based on deep learning methods such as Recurrent Neural Networks (RNNs) and Long Short-Term Memory (LSTM) networks. These studies results showed that the method achieved better performance in identifying the different behavioral states of students. In addition, Chen et al. [8] proposed a behavioral state research method based on decoding analysis, which revealed students’ behavioral patterns and behavioral characteristics in different behavioral states by decoding and analyzing students’ behavioral data during physical education tasks. The results of the study provide strong support for the optimization and improvement of physical education. In addition, Wang et al. [9] applied machine learning methods such as SVM and Random Forest to classify and predict the behavioral states of students during the learning process. The results of the study show that the method is effective in identifying students’ behavioral states and provides a strong support for personalized learning. Chen et al. [10] roposed a Hidden Markov Model approach using Autoencoders for activity recognition of human motion. By introducing self-encoders into the HMM model, automatic learning and representation of human motion features is achieved, thus improving the performance of activity recognition. Lee et al. [11] proposed a Hidden Markov Model approach based on motion vectors and hierarchical pose information for human activity recognition. The accuracy and robustness of human activity recognition is further improved by using motion vectors and hierarchical pose information to represent human motion features. Zhang proposed a method for human activity recognition based on fusion of Hidden Markov Models. By fusing multiple HMM models, the accuracy and robustness of human activity recognition is thus improved [12].

In summary, the behavioral state research based on machine learning and decoding analysis such as HMM has made some progress in the study of human movement state, and the analysis and calculation of relevant data through the analysis of different signals, which provides a theoretical basis for its application in various fields.

### A study of students’ behavioral states in physical education tasks

The study of student behavioral states in physical education tasks is a multidisciplinary field of study involving education, psychology, and exercise science. By observing, analyzing and modeling students’ behavioral states in the physical education classroom, we can gain a deeper understanding of students’ learning processes and learning effects in different teaching environments, providing a scientific basis for optimizing physical education teaching methods and enhancing students’ learning experiences. Recently, research in physical education has gradually introduced machine learning methods to more accurately parse student behavioral states and provide more effective instructional interventions.

In the field of machine learning and deep learning, there are various approaches that have been applied to the study of student behavioral states in physical education tasks. For example, Jiang et al. [13] proposed a method based on a Hidden Markov Model to automate the assessment of students’ skill levels by modeling their action execution processes. Smith et al. [14], on the other hand, used a conditional random field approach to model students’ social interaction behaviors and analyzed the effect of social behaviors on students’ emotional states.

In addition, there are some studies that use deep learning methods such as convolutional neural networks (CNN) and RNN. For example, Li et al. [15] used CNN to extract features from students’ action data in a physical education classroom and constructed an action recognition model to achieve automatic assessment of students’ action execution levels. Li et al. [16] also utilised a long short-term memory (LSTM) housing price prediction model to predict housing price.

In addition, several studies have combined machine learning and deep learning techniques with traditional behavioral observation and questionnaire methods to improve the accuracy and comprehensiveness of student behavioral status. For example, Johnson et al. [17] combined traditional behavioral observation methods and random forest-based machine learning methods to conduct an in-depth study of students’ social behaviors in physical education classrooms and explored the effects of social behaviors on students’ emotional states and learning outcomes. Huang et al. [18], on the other hand, combined questionnaires and deep learning methods to conduct a comprehensive analysis of students’ emotional states and self-efficacy in the physical education classroom to gain insight into students’ behavioral states and learning outcomes.

The study of student behavioral states in physical education tasks is gradually introducing machine learning and deep learning methods to improve the accuracy and comprehensiveness of student behavioral states. These methods provide researchers with powerful tools for gaining a deeper understanding of the patterns of behavioral change, the relationship between behavioral states and learning outcomes, and the possible influences on students in the physical education. Therefore, this paper intends to provide new inspiration for physical education by combining the HMM decoding method, which is widely used in current action sequence research and has low computational load and difficulty, with a basic BP neural network for behavioral state recognition processing analysis.

## A BPNN-HMM-based state analysis and processing model for sports behavior

In the process of physical education, it is difficult to analyze students’ movement status through professional laboratories or professional sports fields due to the unbalanced economic development of each region. Therefore, in practical applications, more emphasis needs to be placed on the applicability of the methods and data used. The current common data in the field of motion analysis mainly includes two major categories of data: image-based and inertial-based. According to the difficulty and scope of practical application, this paper selects inertial data for movement for behavioral state analysis in physical education, and completes the relevant analysis through the bracelets and inertial sensor data worn by students on their hands and ankles.

### BP-NN based motion behavior feature extraction

Human motion state feature extraction based on inertial sensor data is a method to extract human motion state features by signal processing and machine learning techniques using motion data collected from inertial sensors (e.g. accelerometers, gyroscopes, etc.). Among them, BP-NN (Backpropagation Neural Network) is a commonly used machine learning algorithm, which is widely used in pattern recognition, data mining and motion analysis [19]. In this study, we use BP-NN as the main feature extraction algorithm. After completing the filtering and noise reduction of the data, we perform feature extraction of the data by BP-NN and use the sliding window method for analysis. BPNN is a common artificial neural network model and one of the earliest widely used neural network models. Its basic idea is to adjust the weight of the network through the Backpropagation, so that the error between the output result of the network and the expected output result is continuously reduced, and finally reach the convergence state. BP neural networks are commonly used for tasks such as classification, regression, prediction, and recognition. The BP neural network consists of an input layer, a hidden layer, and an output layer. Among them, the input layer receives input signals, the hidden layer is the core of the network, and the selection of the number of neurons and layers has a significant impact on the performance of the network, while the output layer produces the output results of the network. Each neuron in the network has connections with other neurons, and each connection has a weight. The core of the BP algorithm is to optimize the performance of the network by adjusting these weights [20]. The input data are normalized to map different dimensions to the same scale to avoid the uneven influence of different features on the results. The output value of each layer in the neural network is calculated by forward propagation, and the process of calculating from the input layer to the output layer can be expressed as follows.

1 $${a}^{(l)}=g\left({z}^{(l)}\right)$$

Among them, the ${a}^{(l)}$ is the output value of the first $l$ the output value of the layer, and $g(\cdot )$ is the activation function, and ${z}^{(l)}$ is the input value of the $l$ input value of the layer, which can be expressed as

2 $${z}^{(l)}={w}^{(l)}{a}^{(l-1)}+{b}^{(l)}$$

Among them, the ${w}^{(l)}$ is the first $l$ right of the layer, the ${b}^{(l)}$ is the power of the $l$ the bias term of the layer, and ${a}^{(l-1)}$ is the output value of the $l-1$ is the output value of the layer. After completing the forward propagation calculation, the error needs to be calibrated for comparison, and the text uses the mean square error function, which is calculated as shown in Eq. (3):

3 $$E=\frac{1}{2n}\sum\nolimits_{i=1}^{n} {\left({y}_{i}-{\widehat{y}}_{i}\right)}^{2}$$

where $n$ is the sample size, and ${y}_{i}$ is the actual label value, and ${\widehat{y}}_{i}$ is the output of BP-NN. The error is passed backwards from the output layer to the input layer by a backpropagation algorithm, which updates the weights and bias terms of the neural network to reduce the error value. The calculation process of back propagation can be expressed by the following equation:

4 $${\delta }^{(L)}=\left({a}^{(L)}-y\right)\odot {g}^{\mathrm{^{\prime}}}\left({z}^{(L)}\right)$$

5 $${\delta }^{(l)}={\left({w}^{(l+1)}\right)}^{T}{\delta }^{(l+1)}\odot {g}^{\mathrm{^{\prime}}}\left({z}^{(l)}\right)$$

6 $$\frac{\partial E}{\partial {w}^{(l)}}={a}^{(l-1)}{\delta }^{(l)}$$

7 $$\frac{\partial E}{\partial b(l)}={\delta }^{(l)}$$

where ${\delta }^{(L)}$ is the error in the output layer, and ${\delta }^{(l)}$ is the error in the first $l$ the error of the layer, and ${g}^{\prime}(\cdot )$ is the derivative of the activation function, and $\frac{\partial E}{\partial {w}^{(l)}}$ and $\frac{\partial E}{\partial {b}^{(l)}}$ are the partial derivatives of the error function with respect to the weights and bias, respectively. The weights and bias terms are updated using the gradient descent method based on the bias derivatives calculated by back propagation to reduce the error values. The updated formula can be expressed as follows:

8 $${w}^{(l)}={w}^{(l)}-\alpha \frac{\partial E}{\partial {w}^{(l)}}$$

9 $${b}^{(l)}={b}^{(l)}-\alpha \frac{\partial E}{\partial b(l)}$$

where $\alpha$ is the learning rate, the update step of the control weights and bias terms to avoid too fast or too slow updates. After completing the feature screening of BP-NN, i.e., the initial classification of motion to improve the reliability, the data are further processed by HMM sequences. The utilization of Back Propagation Neural Networks (BPNN) for feature extraction in analyzing hand-ring inertial data represents a strategic choice owing to its ability to navigate the complexities inherent in such data sets. BPNNs excel in extracting non-linear features, adapting to diverse data distributions, and automatically learning hierarchical representations, thus facilitating the extraction of relevant information from raw input data. This capability is particularly advantageous in scenarios where manual feature engineering would be impractical or where the optimal features are not readily discernible. Moreover, BPNNs are adept at handling high-dimensional data, mitigating the challenges posed by the curse of dimensionality while maintaining efficiency in data processing. By integrating feature extraction with subsequent analysis tasks into an end-to-end learning framework, BPNNs streamline the overall workflow and enhance the efficiency of data analysis pipelines. Furthermore, BPNNs exhibit strong performance and generalization capabilities, enabling accurate and robust feature extraction for motion sequence analysis. In conclusion, the choice of BPNNs for feature extraction in the context of hand-ring inertial data analysis underscores their versatility, effectiveness, and suitability for capturing meaningful insights from complex motion data, thereby advancing our understanding of students’ motion sequence states and facilitating applications across various domains, particularly in education.

### HMM-based motion sequence classification

The HMM is a classical statistical model for describing probabilistic models with an implicit sequence of states. HMMs are widely used in many fields, including speech recognition, natural language processing, and bioinformatics [21]. HMM is also widely used in state classification of motion sequences for modeling and classifying complex motion sequences. The HMM consists of three sets of parameters: initial state probability (pi), state transfer probability (A) and observation probability (B). where the initial state probability pi represents the probability that the system is in each state at the moment of time step 0. The state transfer probability A represents the probability of transferring to state j in state i at the current moment. The observation probability B denotes the probability of observing observation j in state i at the current moment. The basic idea of HMM is to describe the probabilistic relationship between the implied state sequence and the observed sequence by these three sets of parameters. In HMM, the implicit state sequence is unknown, while the observed sequence is observable. HMM infers the implied state sequence by counting and learning from the observations of the observation sequence. The basic assumption of HMM is Markovianity, i.e., the current state is only related to the previous one. This allows the HMM to handle sequence data with long time dependencies, such as motion sequences. The core problems of HMM include three: the state sequence problem, the observation sequence problem, and the parameter estimation problem [22]. The state sequence problem is to solve the most probable implied state sequence with known parameters and observation sequences of the HMM. The observation sequence problem is to solve the probability of the observation sequence with known parameters and implied state sequence of the HMM. The parameter estimation problem refers to solving the parameters of the HMM given the observed sequence. These three problems can be solved by different algorithms, such as the forward algorithm, the backward algorithm, and the Viterbi algorithm. The forward algorithm is to compute a given observation sequence ${\mathbf{O}}$ and model parameters $\lambda = {\mathbf{A}},{\mathbf{B}},{{\varvec{\uppi}}}$ the first $t$ moment observation is ${\mathbf{o}}_{t}$ and the model is in state $q_{i}$ of the model. The forward algorithm is formulated as follows:

10 $${\alpha }_{t}(i)=P\left({\mathbf{o}}_{1},{\mathbf{o}}_{2},\dots ,{\mathbf{o}}_{t},{q}_{t}={S}_{i}\mid \lambda \right)$$

where $\alpha_{t} (i)$ indicates that at the moment $t$ the model is in the state ${q}_{i}$ and the probability of observing ${\mathbf{o}}_{1} ,{\mathbf{o}}_{2} ,...,{\mathbf{o}}_{t}$ the probability of the model being in the state at the moment, which can be calculated recursively by

11 $${\alpha }_{t+1}(i)=\left[\sum\nolimits_{j=1}^{N} {\alpha }_{t}(j){a}_{ji}\right]{b}_{i}\left({\mathbf{o}}_{t+1}\right)$$

where $a_{ji}$ indicates a transfer from state ${q}_{i}$ transfer to state ${q}_{j}$ the probability of transfer, and $b_{i} ({\mathbf{o}}t + 1)$ denotes the probability of transferring from state ${q}_{i}$ is the probability of observing ${\mathbf{o}}t + 1$ and $N$ denotes the number of states.

The backward algorithm is to compute a given observation sequence ${\mathbf{O}}$ and model parameters $\lambda = {\mathbf{A}},{\mathbf{B}},{{\varvec{\uppi}}}$ the first $t$ moment observation is ${\mathbf{o}}_{t}$ and the model is in state $q_{i}$ of the model. The backward algorithm is formulated as follows:

12 $${\beta }_{t}(i)=P\left({\mathbf{o}}_{t+1},{\mathbf{o}}_{t+2},\dots ,{\mathbf{o}}_{T}\mid {q}_{t}={S}_{i},\lambda \right)$$

where $\beta_{t} (i)$ indicates that at the moment $t$ the model is in the state $q_{i}$ and the probability of observing ${\mathbf{o}}t + 1,{\mathbf{o}}t + 2,...,{\mathbf{o}}_{T}$ the probability of the model being in the state at the moment, which can be calculated recursively by

13 $${\beta }_{t}(i)=\sum\nolimits_{j=1}^{N} {a}_{ij}{b}_{j}\left({\mathbf{o}}_{t+1}\right){\beta }_{t+1}(j)$$

In the process of HMM classification, learning of parameters and inference of states are required. Among them, the parameters can be learned using the EM algorithm (Expectation–Maximization Algorithm), which is used to estimate the parameters of the HMM model from the training data. The EM algorithm is an iterative optimization method that consists of two steps: the E step and the M step. In step E, the posterior probability distribution of the hidden variables, i.e., the probability of being in each hidden state at each moment given the observed data, needs to be estimated. For HMM, this probability can be calculated by both the forward and backward algorithms [23].

**Fig. 1:**
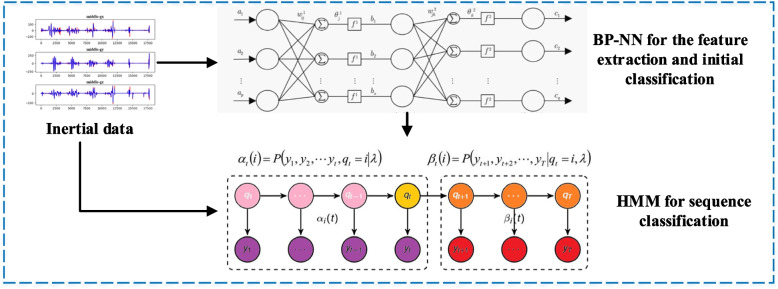
The framework for the proposed motion sequence classification

After completing the above steps, each parameter in the HMM sequence can be estimated to complete the identification of the state sequence, and the proposed framework combing the BPNN and HMM is shown in Fig. 1.

## Experimental results and analysis

According to “A BPNN-HMM-based state analysis and processing model for sports behavior” section, it is difficult to analyze students’ state behaviors directly from video data or from prescribed laboratories because of the wide variation of teaching conditions in the physical education process from region to region. Considering the cost issue and the difficulty of practical application, this paper uses inertial sensor data for the analysis of motion. Considering the purpose of motion recognition, we need to complete the sequence to sequence division, so we use sliding windows to extract action features during the recognition process. Considering that the sampling frequency of the hand ring is approximately 50 Hz, our sliding window length is also set to 50 with a step size of 10, in order to complete the corresponding feature value calculation within each sliding window. In the experiment, we selected the Students’ Behavior dataset (https://zenodo.org/records/8337444), which collected data from the students with four movements of stationary, walking, running and squatting according to the instructions, and extracted the 10-dimensional statistical features like the mean, maximum, minimum of the inertial six-axis data including the acceleration and the angular velocity, so as to complete the corresponding data collection work, the collected data processing process and data analysis. Hand-ring inertial data can capture intricate details of hand movements, including orientation, acceleration, and velocity. This level of granularity enables researchers and educators to analyze students’ motions with high precision, identifying subtle patterns and nuances that may not be apparent through other means. Besides, the hand-ring can minimize the impact of exercise to the greatest extent possible.

Considering the data characteristic, mean filtering is employed to reduce the signal noise.Mean filtering is a common signal processing technique used to remove noise or smooth signals from a signal. It is achieved by calculating the average value of data points within a certain window size in the signal. Mean filtering is usually applied in time series data or image processing, which can be calculated as follows:

14 $$y[n]=\frac{1}{N}\sum\nolimits_{k=-\frac{N-1}{2}}^{\frac{N-1}{2}} x[n+k]$$

where y [n] is the filtered output signal. x [n] is the input signal. N is the window size, usually an odd number, representing the number of data points used to calculate the average in the signal. K is the index in the window, which extends from the center of the window to both sides.

### Data acquisition and model training

**Fig. 2:**
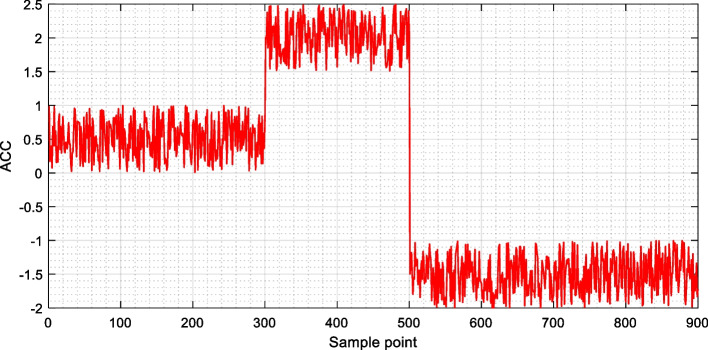
The inertial data in teaching

In order to better analyze the differences of the signals under different actions, a segment of the data was intercepted, and it can be found that the data of the students performing the task at different times has a certain distinguishability, and there is a clear difference in the size of its range, so that it can be distinguished using a machine learning method. The inertial data collected in teaching is shown in Fig. 2.

**Fig. 3:**
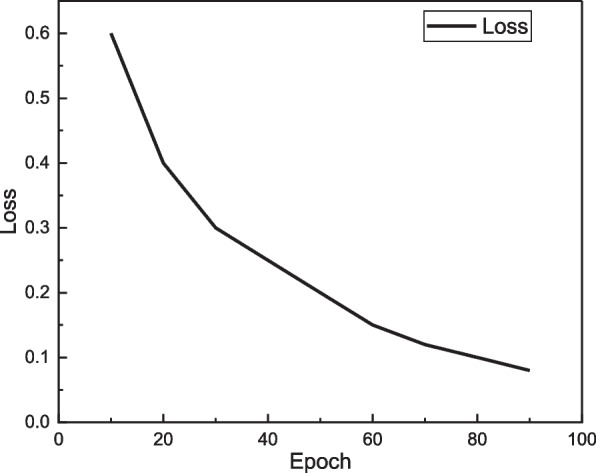
The loss function for the BPNN training

We train the BPNN model before applying the HMM for refinement of action sequences, and its loss function during training is shown in Fig. 3.

After training, the recognition accuracy of our BPNN method on the test set is 87.8%, which can be seen as an acceptable result with high accuracy for most of the recognition problems. The specific recognition rate after adding HMM will be presented in the next section.

### Comparison of motion state recognition

**Fig. 4:**
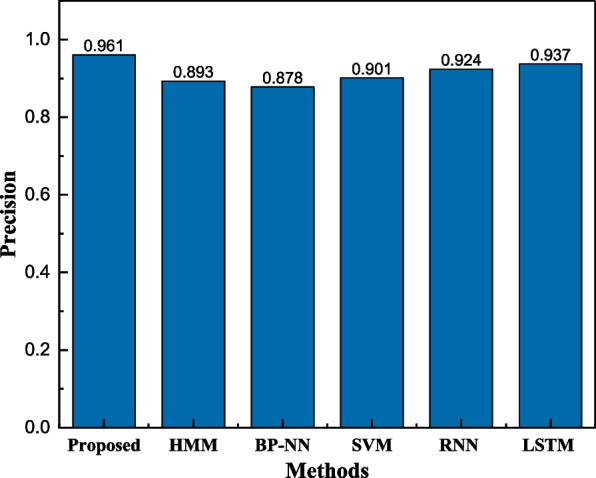
The result for the method comparison

The recognition rate of nearly 90% was obtained by using the BPNN method alone, but we found through further analysis of the classified sequences that there is incoherence in the classified sequences, i.e., there are sudden interruptions in them that misclassify the sequences, a phenomenon that does not exist in practice, so we thought of further refinement by HMM, and the recognition rates of the models under different methods are shown in Fig. 4.

Considering the processing power of time series data, in this paper, the traditional methods are used to compare the results [19]. The proposed framework is combined with the BPNN and HMM, so that the single methods should be compared first. Secondly, as computing ability improves significantly, the simple and classical deep learning methods like RNN and LSTM are also both employed. According to Fig. 4, we can find that the recognition rate of the BPNN-HMM method proposed is significantly better than that of a single method, and better than that of the SVM method, which is more widely used in machine learning. In addition, with the continuous development of deep learning today, we also applied RNN and LSTM methods to compare the proposed methods, and it is easy to find that although the recognition rates of RNN and LSTM methods are over 90%, they are still inferior to BPNN-HMM. On the other hand, the deep learning model is much more complex and computationally consuming than traditional neural network methods due to the more complex models and computational parameters in the building process.

**Fig. 5:**
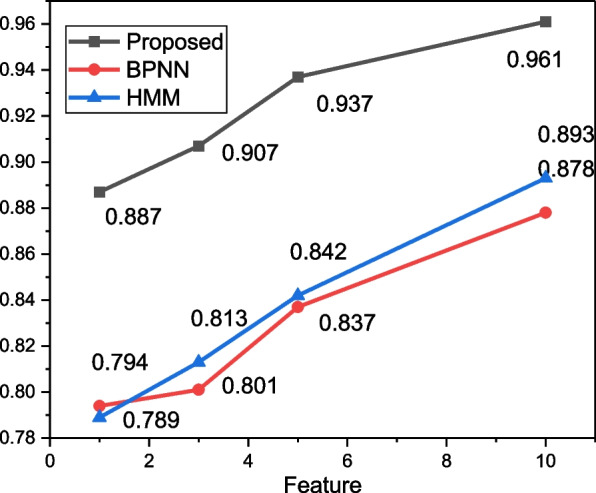
The recognition result under different features

To better illustrate the effectiveness and efficacy of the proposed method, we tested the model under different feature dimensions, and the results are shown in Fig. 5.

According to the data in Fig. 5 we find that with the continuous improvement of the feature dimension, there is a certain improvement of the overall recognition accuracy of the model, so this paper chooses 10-dimensional features, i.e., a total of 60-dimensional input features under six-axis data, which is a better choice.

### Classification effects under different actions

**Fig. 6:**
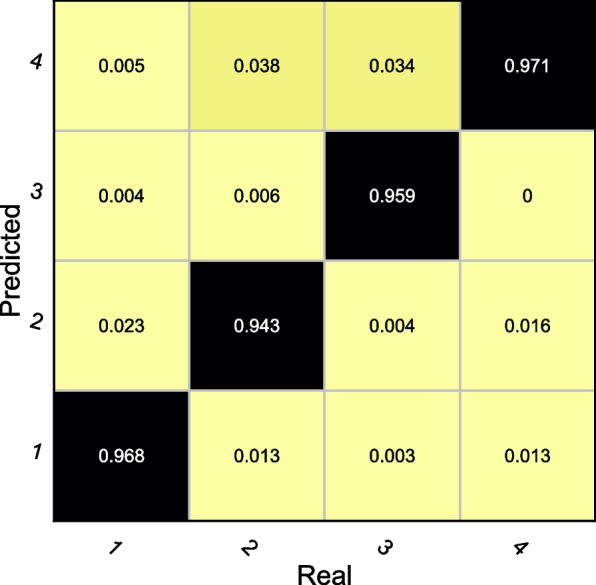
The recognition result of different motions

We performed a refined analysis of the different actions in the sequence to test the recognition features of the model for different actions, and the resulting confusion matrix is shown in Fig. 6.

In Fig. 6, the labeled actions are Stationary, Walking, Running and Squat, and we can find that the model proposed in the paper has a balanced classification of the four types of actions, all around 95%, and the recognition rate of Squat is more than 97%. In the subsequent signal analysis we found that for squat movements, there is a significant increasing trend in the z-axis of the acceleration, which is significantly different from the overall trend change in other movements, so the recognition accuracy for this type of movement is the highest. In the process of selecting research subjects, we selected students from different classes to test the robustness of the model as much as possible. This also indicates in another aspect that for different physical education, its recognition accuracy for various types and groups of students is good.

## Discussion

In this paper, we use a combination of BPNN and HMM to successfully implement an action recognition task in sports. Compared with SVM, LSTM, RNN and other methods, the method proposed has the following advantages. First, BPNN can adjust the weights adaptively, thus improving the accuracy of the model. Compared with traditional machine learning algorithms, BPNN can solve nonlinear problems better [24]. Moreover, the training speed of BPNN is also faster than methods such as LSTM and RNN, which can complete the training of the model faster. Second, HMM can effectively handle time series data, thus improving the identification accuracy of the model. HMM can model time series data and transform it into a series of states to model action sequences. Moreover, the training speed of HMM is also faster than that of methods such as LSTM and RNN, which can complete the training of the model faster [25]. In conclusion, this paper has advantages in recognition accuracy compared with single machine learning or decoding analysis methods, and the accuracy of the proposed combined method is still higher compared with RNN class models which are better at recognizing time-series data, so the model in this paper has considerable advantages in future applications of time series data analysis. Hidden Markov Models (HMMs) enhance the classification capability of sequence models by effectively capturing temporal dependencies and modeling emission probabilities. By considering the transitions between hidden states and the likelihood of observing specific observations given each state, HMMs can discriminate between different classes based on the underlying dynamics of the data. Their robust training and inference algorithms, such as the Expectation–Maximization (EM) algorithm and the Viterbi algorithm, further contribute to their classification performance. HMMs are versatile models applicable to a wide range of sequential data, including speech signals, time series data, and text. Their ability to model complex sequences and handle noisy or incomplete data makes them valuable for various classification tasks in diverse domains. In summary, HMMs provide a powerful framework for enhancing classification capability in sequence modeling, offering a balance between capturing temporal dependencies and modeling emission probabilities to accurately classify sequential data. In summary, Hidden Markov Models (HMMs) excel at capturing temporal dependencies and providing interpretability but may struggle with non-linearity and scalability. Back Propagation Neural Networks (BPNNs) offer robust representation learning but lack explicit temporal modeling. Combining HMMs’ sequential modeling with BPNNs’ representation power can enhance classification by leveraging temporal dynamics and complex feature representations, thereby mitigating each model’s limitations and improving overall performance in sequence modeling tasks.

The use of smart tools needs to be emphasized in physical education. The use of smart tools allows for better assessment of students’ motor skill levels and thus better guidance for training. At the same time, intelligent means can also help teachers to personalize instruction for students and improve their learning effectiveness by providing targeted training according to their actual situation.

Intelligent monitoring of students’ movements through bracelets and videos can timely detect and correct their incorrect movements, improve teaching effectiveness, help students master correct movement skills faster, and reduce the risk of injury. By using bracelets and video monitoring data, the teaching process can be recorded and analyzed, providing teachers with more scientific and intelligent teaching support, and improving teaching efficiency and quality. However, the following points also need to be taken into account when applying smart tools for teaching physical education [26]. First, smart tools are only aids and cannot replace the role of teachers. Teachers need to individualize instruction to help students better understand movement skills. Second, intelligent means also need to be constantly updated and maintained to ensure their accuracy and reliability. Finally, intelligent tools should be integrated with teaching practice and not be detached from practical applications. Only through continuous optimization and improvement in practice can the role of intelligent tools be better utilized.

## Conclusion

This paper presents a methodological framework for analyzing students’ behavioral states in secondary school physical education using hand-ring inertial data decoding and analysis. The proposed method is based on ten types of statistical features of inertial six-dimensional data as model input, and utilizes BPNN network for pre-classification of behavioral states and HMM for further optimization and smoothing of the scored sequences. The results show that the recognition rate of this framework for human movement behavioral state analysis based on bracelet data analysis is over 96%, which is higher than the single use of two methods and RNN/LSTM methods. The combination of BPNN and HMM can achieve the same recognition rate of sequence classification as deep learning methods without increasing the computational load, providing new ideas for future mobile computing and smart sports courses. In the future, intelligent teaching monitoring technology that integrates multi-source data will be widely applied in the field of physical education, bringing more technical support and innovation to physical education.

However, this paper is limited by the convenience of data collection, where only simpler data in physical education were collected and fewer actions were considered, which restricts its large-scale application. Therefore, future research should focus on improving the data collection method, enriching the database content, and enhancing the model’s robustness.

## Data Availability

The dataset emploved in this investigation is made readily available and accessible to interested parties, the data link is as follows: https://zenodo.org/records/8337444
